# Abortion restrictions and female medical school applicants: A retrospective study

**DOI:** 10.1371/journal.pgph.0006436

**Published:** 2026-06-03

**Authors:** Jade Michele Gilchrist, Alexandra C. Istl, Amrit Kirpalani

**Affiliations:** 1 Department of Paediatrics, Schulich School of Medicine and Dentistry, Western University, London, Ontario, Canada; 2 Department of Surgery, Division of Surgical Oncology, Medical College of Wisconsin, Milwaukee, Wisconsin, United States of America; 3 Department of Paediatrics, Division of Nephrology, Children’s Hospital, London Health Sciences Centre, London, Ontario, Canada; The Ohio State University, UNITED STATES OF AMERICA

## Abstract

Following the reversal of Roe v. Wade in 2022, U.S. states enacted divergent abortion policies that may influence women’s educational and career decisions. This study examined whether these policy shifts were associated with changes in female representation among medical school applicants and matriculants across the United States. Using publicly available state-level data from the Association of American Medical Colleges (2018–2025), we conducted a fixed-effects regression analysis comparing trends in states with abortion protections (Expanded/Protected) versus those with restrictive or absent protections (Hostile/Not Protected). Forty-five jurisdictions were included after excluding six with incomplete data. Across the study period, the proportion of female applicants to medical school increased nationally by 5.3 percentage points. However, after reversal of Roe v. Wade, this growth was significantly slower in Hostile/Not Protected states than in Expanded/Protected states (β = -0.58 percentage points per year; 95% CI -1.14 to -0.025; p = 0.041). No statistically significant difference was observed in female matriculants between policy groups (β = 0.68; 95% CI -0.70 to 2.06; p = 0.332). Parallel-trend testing confirmed similar trajectories across groups before the reversal of Roe v. Wade, supporting that divergence emerged after policy change. Subgroup analyses within restrictive states revealed no differential effects by type of abortion ban (trigger, pre-viability, or reason-based). These findings suggest that restrictive reproductive policies may be subtly reshaping women’s professional pathways even at the earliest stages of the physician workforce pipeline. Although absolute differences remain modest, sustained disparities in application growth could have long-term implications for gender equity and healthcare access, particularly in states already facing physician shortages. Understanding how reproductive autonomy intersects with higher education and workforce planning is essential to ensuring equitable representation in medicine and maintaining a resilient healthcare system.

## Introduction

Since its enactment by the Supreme Court in in 1973, Roe v. Wade affirmed and protected the constitutional right to abortion in the United States [1]. However, just under fifty years later, the 2022 Dobbs v. Jackson Women’s Health Organization case led to the overturning of this decision, bringing an end to federal protection of abortion rights. With each state granted the power to enact individual abortion laws [1], there was a rapid and fragmented policy response across states. Several states enacted total or near-total abortion bans. Among these, some had “trigger bans” in place to immediately criminalize the actions of individuals either seeking or providing abortions once Roe v. Wade was overturned, while others continued to permit legal abortions with certain restrictions and eligibility requirements. Other states enacted legislation to preserve and protect abortion rights, with some creating additional policies to expand access to abortion [2]. This fractured legal landscape has renewed scholarly attention towards the intersection of reproductive autonomy with the broader issues of equity, accessibility, and educational opportunity.

Recent studies have demonstrated how abortion access continues to impact women’s socioeconomic outcomes. Denial of abortion care is associated with higher likelihood of poverty and lower likelihood of full-time employment amongst women, compared to those able to pursue and receive abortion care [3]. Conversely, regions with accessible, less restrictive abortion care correlate to a higher likelihood of graduating from college, a greater income, and economic stability [4].

Educational decisions—particularly among women—are deeply influenced by reproductive rights and autonomy, reproductive healthcare availability, and related state-level policies. Access to contraception – specifically, oral contraceptive pills (OCPs) – directly allowed for greater career investment among women since its availability to young, unmarried women in the 1960s [5]. Moreover, there is an association between improved reproductive health access for women and educational and economic empowerment [6,7], and prior research has linked abortion access to educational attainment and professional planning. Anticipatory abortion bans and inaccessibility in American states have been associated with a decline in female college applicants to colleges in these states [8].

Medical schools are critical gateways to the healthcare workforce, making the demographic makeup of applicants and matriculants incredibly important as it helps shape the future of care delivery and workforce diversity [9–11]. There is a strong connection between medical education and workforce contribution as physicians tend to practice in the state or region where they are trained [12]. Given the prolonged and intensive demands of medical training, concerns about reproductive autonomy and in-state policies may have particularly significant implications for women’s educational and career decisions. These shifts are particularly poignant in regions already experiencing provider shortages [13]. Therefore, trends in who applies to medical school – and where – are consequential for local healthcare landscapes.

However, while associations with access to reproductive care have been studied in undergraduate populations [8], little is known about how such policy shifts may affect female representation in graduate and professional education, including medical school. This study examines whether the reversal of *Roe v. Wade* was associated with differential trends in the proportion of female medical school applicants and matriculants in states with restrictive versus protective abortion policies.

## Methods

### Ethics statement

All data used in this study were publicly available through the AAMCS and fully de-identified. This study was exempt from Research Ethics Board approval in accordance with Article 2.2 of the Tri-Council Policy Statement (TCPS2).

### Study design, study population and data source

We conducted a retrospective cohort study examining trends in the gender composition of medical school applicants and matriculants across the United States over an eight-year period, from 2018 to 2025. This analysis used annual, jurisdiction-level data from the Association of American Medical Colleges (AAMC), made publicly available in their “Data & Reports” section [14]. These reports include aggregate data on all individuals who applied to and matriculated into U.S. medical schools via the American Medical College Application Service (AMCAS). The data are anonymous, publicly accessible, and contain no personally identifiable information.

The dataset was accessed on March 15, 2025. At that time, the most recent application and matriculation data (covering the 2025 entering class) were available. All analyses were performed at the jurisdictional level (i.e., state or Washington, D.C.), with state-year units of observation for both applicants and matriculants.

### Abortion policy classification

States and jurisdictions were categorized based on their abortion laws using policy classifications from the Center for Reproductive Rights (2). These categories were used to stratify states into two major groups for analysis:

- **Expanded/Protected**: States that either codified the right to abortion into law (Expanded) or maintained protective policies without further expansions (Protected). These jurisdictions demonstrated a consistent legal commitment to preserving reproductive autonomy.
- **Hostile/Not Protected**: States that implemented restrictive abortion laws following the Supreme Court’s overturning of *Roe v. Wade*, including trigger bans, gestational limits, or reason-based prohibitions (Hostile), and those that lacked formal legal protections (Not Protected).

The classification is summarized in Table 1 and geographically summarized in Table 2. State assignments remained fixed over the study period for consistency, reflecting their status at the time of Roe’s reversal.

**Table 1:** Definitions of State Category Based on Abortion Laws.

Category	Definition
Expanded	Statutory protections ensuring abortion access and, in some cases, additional measures facilitating reproductive healthcare.
Protected	Maintain abortion rights without additional legislative expansions beyond previous protections.
Not Protected	Do not have explicit abortion protections in place but also did not implement immediate trigger bans or highly restrictive measures following Roe’s overturning.
Hostile	Enacted significant restrictions on abortion, including pre-viability bans, gestational limits, and trigger bans that took effect after Roe’s repeal.

**Table 2:** States Categorized by Abortion Laws.

Category	States	
Expanded	California	
	Connecticut	
	Hawaii	
	New York	
	Oregon	
	Vermont	
	Washington	
Protected	Florida	New Jersey
	Iowa	Nevada
	Illinois	Rhode Island
	Kansas	*[Alaska]*
	Massachusetts	*[Delaware]*
	Maryland	*[Maine]*
	Minnesota	*[Montana]*
Not Protected	Colorado	
	DC	
	New Hampshire	
	New Mexica	
	Virginia	
	*[Wyoming]*	
Hostile	Alabama	Nebraska
	Arkansas	Ohio
	Arizona	Oklahoma
	Georgia	Pennsylvania
	Indiana	South Carolina
	Kentucky	South Dakota
	Louisiana	Tennessee
	Michigan	Texas
	Missouri	Utah
	Mississippi	Wisconsin
	North Carolina	West Virginia
	North Dakota	*[Idaho]*

*[Excluded].

### Study period definitions

To better examine trends in relation to the Supreme Court decision, we divided the study period into three key phases:

- **Pre-Roe (2018–2021)**: The period before any formal judicial changes.
- **Anticipatory (2021–2022)**: The interval during which speculation and public discourse in the potential overturn of *Roe v. Wade* intensified, notably after the leaked draft opinion, may have influenced behavior before any formal changes were made be the Supreme Court.
- **Post-Roe (2022–2025)**: The period following the Supreme Court’s formal decision in *Dobbs v. Jackson Women’s Health Organization*, which overturned *Roe v. Wade*.

### Statistical analysis

We used a state and year fixed-effects regression model to analyze trends in the proportion of female medical school applicants and matriculants pre-Roe and post-Roe. This model accounts for:

- **State fixed effects**, which control for time-invariant differences across states (e.g., culture, geography, or baseline political leanings).
- **Year fixed effects**, which account for national-level temporal trends (e.g., changes in the popularity of medicine, or effects of the COVID-19 pandemic).

This model accounts for time-invariant state differences and national temporal trends and allows for a more robust estimate of how abortion policy may influence applicant behavior beyond general temporal or regional variation.

The outcome variable was the annual proportion of applicants or matriculants identifying as female in each jurisdiction. For applicants, we also calculated the absolute change in female representation from 2018 to 2025.

A parallel trends analysis was conducted to ensure that trajectories in female applicants pre-Roe were similar across policy groups. A separate subgroup analysis was conducted within the Hostile/Not Protected states to determine if different types of bans (e.g., pre-viability vs. trigger bans) yielded divergent effects..

## Results

Forty-five U.S. jurisdictions (44 states and Washington, D.C.), with 17 classified as Expanded/Protected and 28 as Hostile/Not Protected, were included. Six states (Alaska, Delaware, Idaho, Maine, Montana, Wyoming) were excluded due to missing data.

### National trends in female representation

Across all years, the average annual number of total applicants was 12,529.5 (SD = 1,618.54) with 6,683.6 female applicants (52.93%). Across the study period (2018–2025), the national proportion of female applicants increased by 5.28 percentage points, equating to an average annual increase of approximately 646 female applicants. This increase was consistent with broader trends in medical education toward gender parity. A similar trend was also observed in female matriculants, although with more variability across jurisdictions.

### Regression results: Applicants

The fixed-effects regression model revealed that, post-Roe, there was an overall increase in the proportion of female applicants nationally by +4.07 percentage points (95% CI: 3.69–4.46; p < 0.001), representing approximately 498 additional female applicants per year.

However, the rate of increase in Hostile/Not Protected states was significantly attenuated compared to Expanded/Protected states. Specifically, the growth in female applicants was reduced by an average of 0.58 percentage points per year (95% CI: -1.14 to -0.025; p = 0.041), corresponding to approximately 71 fewer female applicants annually in these states relative to what would have been expected under neutral trends. This suggests that the proportion of female applicants grew at a slower rate in restrictive states relative to Expanded/Protected jurisdictions.

### Regression results: Matriculants

In contrast, no statistically significant post-Roe difference in the proportion of female matriculants was observed between policy groups. The change in matriculant proportions in Hostile/Not Protected states compared to Expanded/Protected states was + 0.68 percentage points (95% CI: -0.70 to 2.06; p = 0.332), suggesting that while fewer female applicants emerged from restrictive states, those who did apply had similar matriculation rates.

### Parallel trends analysis

**Fig 1:**
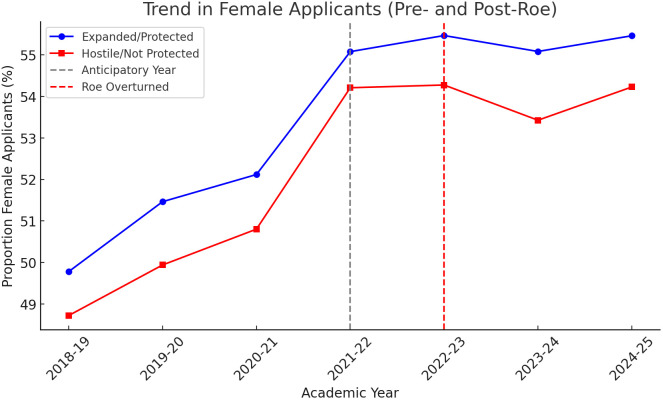
Parallel Trends of Female Applicants in States with and without abortion protection.

Pre-Roe (2018–2021), the trajectories of female applicants in both Expanded/Protected and Hostile/Not Protected jurisdictions were similar, with no statistically significant divergence, supporting the assumption that post-Roe, differences were due to policy shifts rather than pre-existing divergence. Fig 1 displays the parallel trend lines during the pre-Roe period, followed by a visible divergence in female applicant proportions in the post-Roe period.

### Subgroup analysis: Hostile/not protected states

Within the group of Hostile/Not Protected states, we further analyzed whether the specific type of abortion restriction had a differential effect on female applicant trends. States were classified into subtypes including:

- **Trigger bans**: Automatic laws activated after Roe’s reversal
- **Pre-viability bans**: Restricting abortion before fetal viability
- **Reason bans**: Restricting abortion based on fetal anomaly, sex, or race

No statistically significant differences in female applicant trends post-Roe were found across these subgroups (all p-values > 0.05).

## Discussion

Our findings indicate the reversal of *Roe v. Wade* may have impacted the relative growth in female medical school applicants in states with abortion restrictions (*Hostile/Not Protected*). While the nation-wide trends demonstrate and overall increase in female applicants to medical school with each application year, states without reproductive health protections experienced a significantly slower rate of growth. This is an important observation and potential indicator of more significant future shifts in the physician workforce in these states.

Although the absolute difference between abortion protective and restrictive states is modest, seemingly small shifts in applicant pools can have outsized long-term effects – especially if trends continue. This is particularly important in fields like medicine, where entry is highly competitive and training is resource intensive. A slight dip in the number of female applicants could translate into a more substantial shortage of female physicians, and greater deficits in specialities like obstetrics and gynecology where there are higher proportions of female providers [15].

We must acknowledge that our findings on female applicant trends were not accompanied by a corresponding shift in female matriculants to medical school. This may suggest that female applicants are not opting out of medicine altogether but instead are adjusting their strategies for pursuing medical education. For example, female applicants choosing to avoid states with restrictive policies, may be both applying to and matriculating in more states with protective reproductive legislation.

A growing body of literature is highlighting how political climates influence behavior, and shape access to education. Our results align with broader literature showing that young women consider reproductive policies and autonomy when making decisions about pursuing college undergraduate programs [16]. This notion extends to professional schools, which demand long-term planning and personal sacrifice. For these students, the perceived stability of reproductive rights may influence application decisions in subtle but measurable ways.

This trend is not limited to medical school applications. Emerging survey data is starting to identify that many students do not want to train in abortion-ban or abortion-restrictive states. For example, in recent survey by Mermin-Bunnell et al. [16], a majority of medical students reported they were unlikely to pursue residency training programs in a state with abortion restrictions, and female respondents were more driven in this regard than male respondents. As physicians tend to practice in the state or region where they complete their medical school and residency [12], this suggests the impact of state policies will continue to be felt as students transition into the physician workforce. Additional links between state political climates and career decision-making have also been shown through practice location preferences amongst physicians [17].

In the context of gender equity in the physician workforce, a longstanding disparity between male and female physicians in the United States exists [18]. While the proportion of female matriculants to medical school has grown in recent years, there continues to be a gap in leadership, administrative, and academic roles. Importantly, states with abortion restrictions are often the same jurisdictions already facing critical workforce shortages. Recent studies show the distribution of training and specialty decisions are changing, with a notable decline in interest and pursuit of obstetrics and gynecology, a female-dominated specialty, in states with stricter abortion policies [13,15,19]. If such trends in barriers to medical education for women persist, and fewer women apply to medical school in these states or choose to practice there afterwards, the downstream impacts could cause further perpetuation of gender inequities in the physician workforce, medical leadership and legislation, augmenting regional disparities.

Combined, the literature informs of impending geographic and gender maldistributions in healthcare access. Further, policy shifts that dissuade female applicants today may also impact future generations, which will intensify disparities over time. Beyond workforce composition, these trends carry important implications for population health [20]. Regions experiencing attenuated growth in female medical school applicants may face compounded challenges in access to care, particularly for reproductive, maternal, and preventive health services. Female physicians are more likely to practice in primary care and to care for underserved populations, and greater gender concordance between patients and physicians has been associated with improved communication, trust, and certain health outcomes [12,13,20]. Consequently, policies that indirectly deter women from entering local training pipelines may exacerbate existing inequities in healthcare availability and quality, particularly in states already facing physician shortages.

Gender-based differences in application behavior should also be understood within broader structural contexts. Political climate, socioeconomic background, and race/ethnicity shape educational opportunity and perceptions of safety, belonging, and long-term viability within a state. Restrictive reproductive policies may therefore function as a visible signal of broader sociopolitical environments that differentially affect marginalized groups. Although our analysis focuses on gender, these intersecting dimensions likely compound one another, and future work should examine how abortion policy interacts with race, class, and geography to influence medical career trajectories. Conceptually situating reproductive policy within a wider framework of structural determinants of educational access may help clarify how state-level legislation contributes to stratification within the physician pipeline.

Our results, while preliminary, suggest the unfolding political climate surrounding reproductive health post-reversal of Roe v. Wade is impacting the career decision-making of future physicians in the United States, with potential consequences over the long-term on its healthcare system. From a higher education perspective, this study highlights the need to understand how policy environments shape who feels able or willing to pursue professional degrees. Admissions offices and policymakers alike should be attentive to these dynamics, as they influence both access and diversity in education and in the workforce.

There are many limitations to this study. While the current analysis shows a correlation between declining growth in female medical school applicants in abortion-restrictive states post-*Roe v. Wade*’s reversal, we cannot be certain of causal or long-term effects. Moreover, this study is restricted to medical school applicants and does not explore further milestones in the medical career pathway. Finally, educational and career decisions are influenced by an individual’s intersectionality and lived experiences, which the objective data in this study cannot capture.

Future research should examine whether these trends persist over time as political climates continue to develop and change, and explore additional factors, such as specialty selection, residency applications, and retention of female physicians in states with restrictive reproductive policies. There is also a need for qualitative exploration of female applicants’ perspectives that shape their motivations, concerns and decisions on where and why they apply and matriculate to medical school.

## Conclusion

Reproductive policy shifts may be subtly but meaningfully influencing women’s pursuit of medical education. The attenuated growth in female applicants in states with abortion restrictions suggests that reproductive autonomy is not only a health issue, but also a factor shaping participation in highly skilled professional training pathways. While the absolute differences observed are modest, sustained divergence in applicant growth has the potential to compound over time, contributing to gendered and geographic maldistributions within the physician workforce.

These findings matter beyond medical school admissions and may ultimately influence local access to care, availability of reproductive and primary care services, and broader health equity. Our results underscore how legislative decisions outside the education sector can reverberate through workforce pipelines in ways that are difficult to reverse.

From a higher education and policy perspective, this study highlights the importance of considering reproductive autonomy as a structural determinant of educational access. Ongoing monitoring of application, training, and practice patterns, alongside qualitative work exploring applicants’ lived experiences and decision-making, will be essential to understanding the long-term consequences of the post-Roe policy landscape. Ensuring a resilient, equitable healthcare system requires attention not only to who enters medicine, but also to the social and political conditions that shape who feels able to pursue it..

## Supporting information

S1 Data Raw Combined Dataset. (XLSX)
